# Three Gorges Dam: the changing trend of snail density in the Yangtze River basin between 1990 and 2019

**DOI:** 10.1186/s40249-023-01095-y

**Published:** 2023-04-28

**Authors:** Yanfeng Gong, Yixin Tong, Honglin Jiang, Ning Xu, Jiangfan Yin, Jiamin Wang, Junhui Huang, Yue Chen, Qingwu Jiang, Shizhu Li, Yibiao Zhou

**Affiliations:** 1 Fudan University School of Public Health, Building 8, 130 Dong’an Road, Xuhui District, Shanghai, 200032 China; 2 Key Laboratory of Public Health Safety, Fudan University, Ministry of Education, Building 8, 130 Dong’an Road, Xuhui District, Shanghai, 200032 China; 3 Fudan University Center for Tropical Disease Research, Building 8, 130 Dong’an Road, Xuhui District, Shanghai, 200032 China; 4 School of Epidemiology and Public Health, University of Ottawa, 600 Peter Morand Crescent, Ottawa, ON K1G 5Z3 Canada; 5 National Institute of Parasitic Diseases, Chinese Center for Disease Control and Prevention, Shanghai, 200025 China; 6 Chinese Center for Tropical Diseases Research, NHC Key Laboratory of Parasite and Vector Biology, Shanghai, 200025 China

**Keywords:** *Oncomelania hupensis*, Snail, Three Gorges Dam, Environmental change, Long-term trend, Schistosomiasis japonica

## Abstract

**Background:**

The area of *Oncomelania hupensis* snail remains around 3.6 billion m^2^, with newly emerging and reemergent habitats continuing to appear in recent years. This study aimed to explore the long-term dynamics of snail density before and after the operation of Three Gorges Dam (TGD).

**Methods:**

Data of snail survey between 1990 and 2019 were collected from electronic databases and national schistosomiasis surveillance. Meta-analysis was conducted to estimate the snail density. Joinpoint model was used to identify the changing trend and inflection point. Inverse distance weighted interpolation (IDW) was used to determine the spatial distribution of recent snail density.

**Results:**

A total of 3777 snail survey sites with a precise location of village or beach were identified. For the downstream area, snail density peaked in 1998 (1.635/0.11 m^2^, 95% *CI:* 1.220, 2.189) and fluctuated at a relatively high level before 2003, then declined steadily from 2003 to 2012. Snail density maintained lower than 0.150/0.11 m^2^ between 2012 and 2019. Joinpoint model identified the inflection of 2003, and a significant decreasing trend from 2003 to 2012 with an annual percentage change (APC) being − 20.56% (95% *CI:* − 24.15, − 16.80). For the upstream area, snail density peaked in 2005 (0.760/0.11 m^2^, 95% *CI:* 0.479, 1.207) and was generally greater than 0.300/0.11 m^2^ before 2005. Snail density was generally lower than 0.150/0.11 m^2^ after 2011. Snail density showed a significant decreasing trend from 1990 to 2019 with an APC being − 6.05% (95% *CI:* − 7.97, − 7.09), and no inflection was identified. IDW showed the areas with a high snail density existed in Poyang Lake, Dongting Lake, Jianghan Plain, and the Anhui branch of the Yangtze River between 2015 and 2019.

**Conclusions:**

Snail density exhibited a fluctuating downward trend in the Yangtze River basin. In the downstream area, the operation of TGD accelerated the decline of snail density during the first decade period, then snail density fluctuated at a relatively low level. There still exists local areas with a high snail density. Long-term control and monitoring of snails need to be insisted on and strengthened.

**Graphical Abstract:**

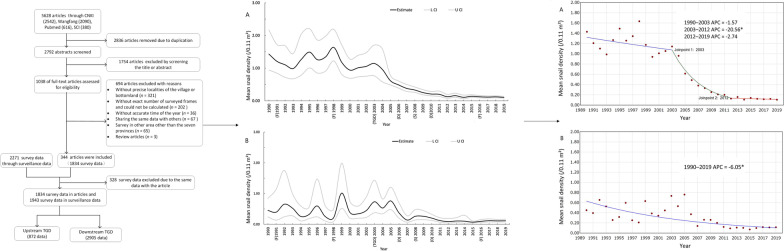

**Supplementary Information:**

The online version contains supplementary material available at 10.1186/s40249-023-01095-y.

## Background

Schistosomiasis is water-borne helminthiasis that affects more than 250 million people with approximately 800 million at risk worldwide [1–3]. In China, schistosomiasis caused by *Schistosoma japonicum* is a zoonotic parasitic disease transmitted by water contact [4]. *Oncomelania hupensis* snail is responsible for the transmission of schistosomiasis japonica [5]. Over the past 70 years, China has implemented comprehensive schistosomiasis control measures focusing on snail control (1950–1985), large-scale chemotherapy (1985–2004) and infectious source control (2005–), and made remarkable achievements [6, 7]. Currently, the major endemic areas concentrate on the seven provinces of the Yangtze River basin, which can be divided into the hilly epidemic area at the upstream of The Three Gorges Dam (TGD) and epidemic area dominated lake and marshland at the downstream of TGD [8].

TGD began filling with water in 2003, and the water level subsequently rose to the heights of 135 m (2003–2005), 156 m (2006–2007), and 175 m (2008–2012) [9]. The impoundment of the TGD has a long-term influence on the water level and the ecological environment in the middle and lower reaches of the Yangtze River [10], which make up approximately 96.8% of the *O. hupensis* habitats in China [11]. The increase of extreme weather events over the past decades such as floods in 1991, 1998 and 2016, droughts in 2006 and 2010, snow storm in 2008, undoubtedly affected the distribution of snail population. The impact of environmental change on snail distribution has invoked heated attention. Numerous studies have been conducted to explore the impact of environmental changes on the snail distribution and the spread of schistosomiasis [12–20]. However, little is known about their impact on the dynamics of snail density [12–20].

The abundance of snails can promote schistosomiasis transmission, so understanding the dynamics and spatial distribution of snail density is critical for schistosomiasis risk assessment and snail control [21–23]. However, most previous studies on the distribution of snail focused on the suitability probability, and rarely quantified the snail density [25–29]. In this study, we conducted a meta-analysis to study the long-term dynamics of snail density from 1990 to 2019 by collecting snail survey data with precise location of the village or beach from multiple data sources, and spatial interpolation to analyze the spatial characteristics of recent snail density.

## Methods

### Literature review

The articles associated with snail density from January 1, 1990 to November 21, 2022 were retrieved from four electronic bibliographic databases, i.e., China National Knowledge Infrastructure (CNKI, https://www.cnki.net/), Wanfang Database (https://www.wanfangdata.com.cn/), PubMed/MEDLINE, and Science Citation Indexed Expanded (SCIE). The search terms were: “schistosomiasis or snail or *Oncomlania hupensis*” and “survey or surveillance or monitor or density”. Relevant literature was also checked manually by searching the reference lists. The criteria of articles included in our study were: (1) a study was conducted in the seven provinces (downstream of the TGD: Hunan, Hubei, Jiangxi, Anhui and Jiangsu; upstream of the TGD: Sichuan and Yunnan); (2) the survey time was clearly reported, at least to a specific year; (3) specific survey location was provided, specifically to village or beach; (4) the number of survey frames and the number of living snails were included, or it can be calculated by the formula (living snail density = the number of living snail/the number of surveyed frames); (5) non-review articles; (6) Full text is available. If any one of the above standards is not met, it shall be excluded. If two articles published the same research data, the article with less information was excluded. There are few articles reporting the snail survey after 2019 to date; therefore, our research focused on the period from 1990 to 2019.

### Data extraction

All research documents were imported into the document management software NoteExpress3.7 (Beijing Aegean Hailezhi Technology Co., Beijing, China) and duplicate records were eliminated. Two researchers independently conducted a preliminary screening of each identified title and abstract, and then read the full text for a second screening. Relevant information for each study was extracted using pre-designed Microsoft Excel tables, including references, survey time (year), study location (province, village or beach), the number of surveyed frames, the number of living snails, and density of living snails. Similar information was also collected from the national schistosomiasis surveillance for the period from 1990 to 2019.

### Data analysis

#### Meta-analysis

Snail density and its 95% confidence interval (*CI*) were integrated for the upstream and downstream of the TGD, respectively, for the whole study period (1990–2019), and subgroup analysis was conducted according to the survey year to obtain the annual density of snails. The heterogeneity between studies was assessed using Cochran’s *Q* (chi-square) test and quantified by Higgins inconsistency statistic (*I*^*2*^) [30]. *I*^*2*^ indicates whether a difference between studies is caused by heterogeneity rather than chance, and the values of 25%, 50% and 75% correspond to low, medium, and substantial heterogeneity [31]. When there was substantial heterogeneity (*I*^*2*^ > 50%), a random effect model was used to combine snail density estimates; otherwise, a fixed effect model was used [32]. All the meta-analyses were performed with Stata17.0 (Stata Corp., College Station, Texas, USA).

#### Changing trend of snail density

Joinpoint model was used to explore the changing trend of snail density for the period between 1990 and 2019 (Joinpoint Regression Program 4.8.0.1, National Cancer Institute, Rockville, MD, USA). Logarithmic linear model for trend was selected to calculate the annual percentage change (APC) and its 95% *CI* [33]. APC is a dimensionless relative number, which only reflects the direction and speed of the trend change. APC < 0 indicates that the snail density decreases with time, APC > 0 indicates that the snail density increases with time [34]. A replacement test was used to determine the number of connection points and the position of each connection point, and to test whether the trend of snail density for each time segment was statistically significant.

#### Surface interpolation to estimate mean snail density

In order to explore the spatial characteristics of recent snail density, the survey data between 2015 and 2019 were used. Snail density at the same point was averaged for the period between 2015 and 2019, and then inverse distance weighted (IDW) interpolation was used to estimate the mean density of snails. IDW is a deterministic spatial interpolation method, which takes the spatial distance between object points as a weight [35]. The closer the distance is, the greater the weight is [35]. The formula for calculating the estimated average snail density D at the interpolation point is as follows [36]:

$$D={\sum }_{i=1}^{n}{\omega }_{i}D({s}_{i})$$

$n$ represents the number of known snail points; $D({s}_{i})$ is mean snail density at the snail point ${s}_{i}$; ${\omega }_{i}$ is Weight of sample point ${s}_{i}$. Weight ${\omega }_{i}$ is calculated as follows [36]:

$${\omega }_{i}=\frac{{d}_{i}^{-P}}{\sum_{i=1}^{n}{d}_{i}^{-P}}$$

${d}_{i}$ is the distance between the prediction snail point $s$ and the known snail point ${s}_{i}$.$P$ is the power of the distance. The larger the exponent value, the greater the influence of the closer snail sample points on the result. The interpolation analysis was performed with ArcGIS 10.2 software (Esri, Redlands, CA, USA).

## Results

### Sites of snail surveys included in the current analysis

**Fig.1:**
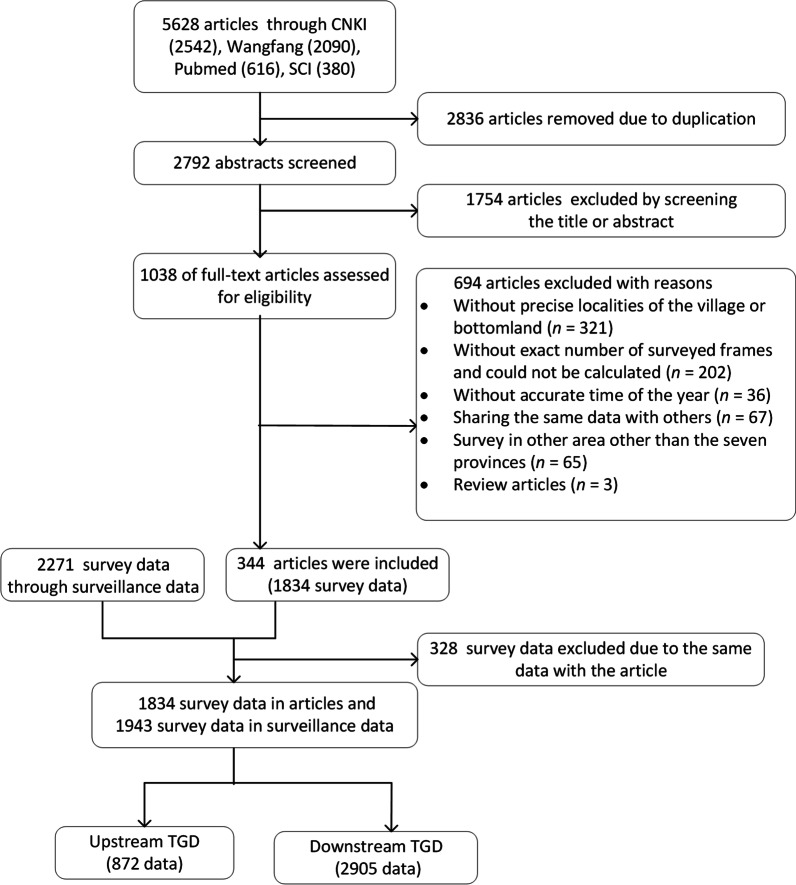
Flow chart of search and selection of articles

A total of 5628 articles were identified from the 4 electronic databases. Of them, 2836 articles were removed due to duplication, 1754 were irrelevant by screening the title or abstract, and 694 were excluded after screening 1038 full-text articles (Fig. 1). In addition, 2271 survey sites in the 7 provinces were identified from the national surveillance data, and 328 of them were excluded due to the same data sites identified from the research articles (Fig. 1). Finally, the analysis included 1834 independent survey sites from research articles and 1943 independent survey sites from the surveillance for the period between 1990 and 2019, and 872 were located at the upstream area of TGD and 2905 at the downstream of the TGD. The location of these independent survey sites is presented in Additional file 1: Fig. S1.

### Changing trend of snail density

**Fig.2:**
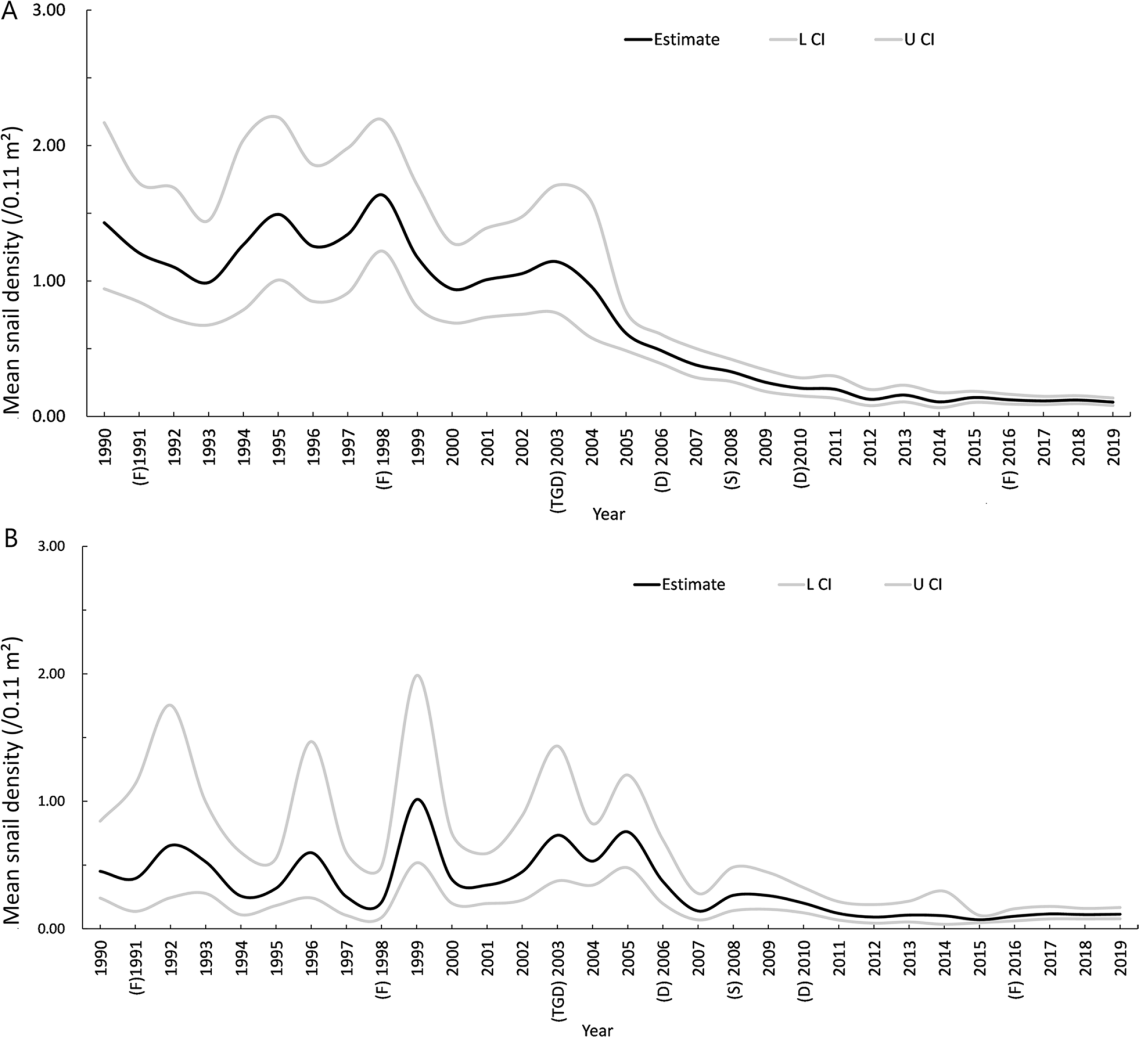
Estimation of the mean density of *O. hupensis* snails between 1990 and 2019. **A** The downstream area of TGD; **B** the upstream area of TGD). *F* Flood; *TGD* Three Gorges Dam;* D* Drought;* S* Snow*; L CI* Lower limit of the 95% confidence interval; *U CI* Upper limit of the 95% confidence interval

There was heterogeneity between studies (upstream area: *Q* = 3.4e + 06, *P* < 0.001, *I*^*2*^ = 99.99%; downstream area: *Q* = 1.2e + 07, *P* < 0.001, *I*^*2*^ = 99.99%), a random effect model was used to estimate the density of snails. The overall density in the downstream area was 0.353/0.11 m^2^ (95% *CI:* 0.329, 0.378) compared with 0.180/0.11 m^2^ (95% *CI:* 0.159, 0.203) in the downstream area. The annual snail density showed a decreasing trend for both areas (Fig. 2).

**Fig. 3:**
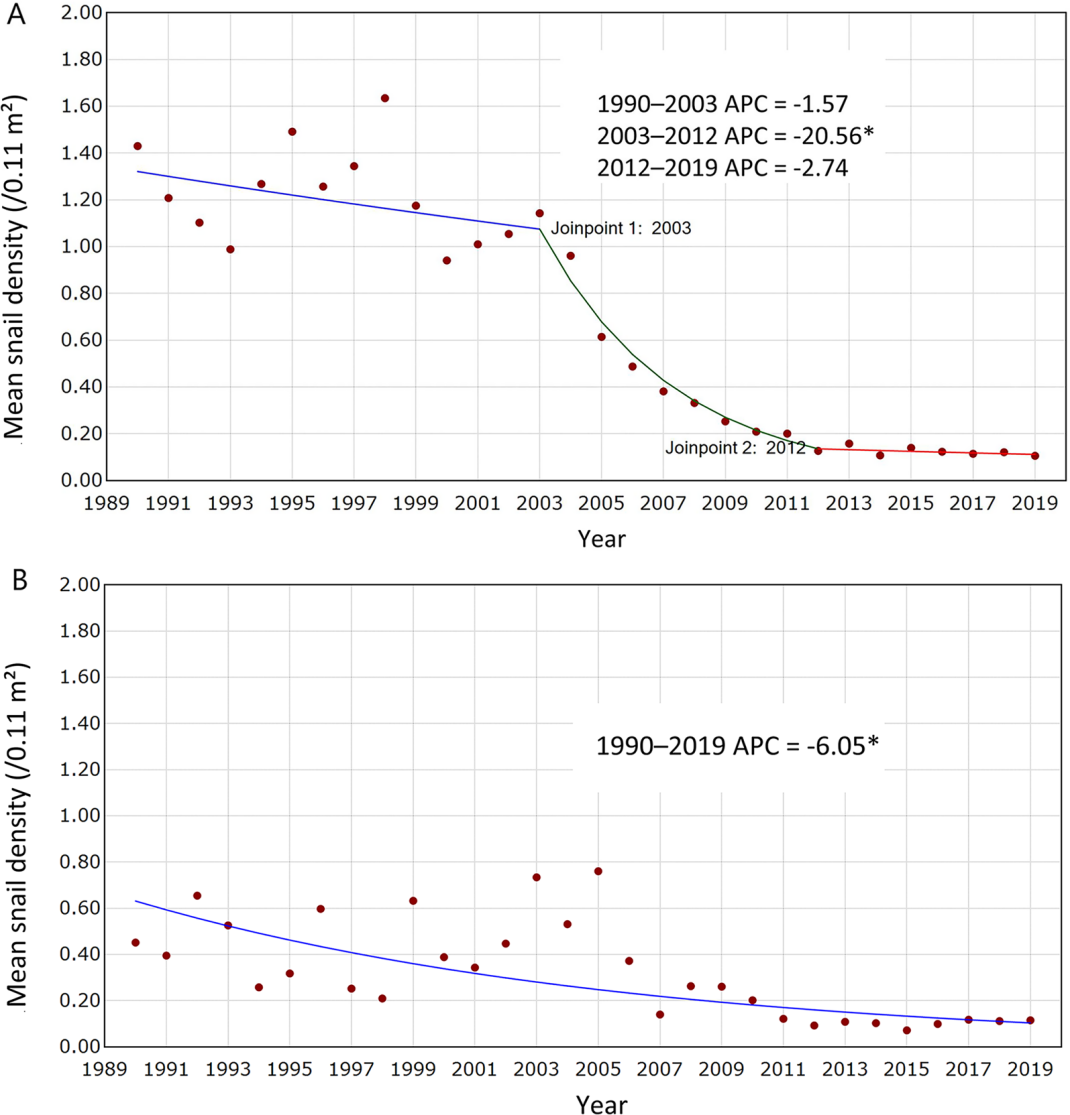
Changing trend of *O. hupensis* snail density between 1990 and 2019. **A** The downstream area of TGD; **B** the upstream area of TGD). * denotes *P* < 0.05; *APC* Annual percentage change

For the downstream area of the TGD (i.e., the lake and marshland regions) (Fig. 2A), the snail density declined significantly from 1.430/0.11 m^2^ (95% *CI:* 0.943, 2.169) in 1990 to 0.106/0.11 m^2^ (95% *CI:* 0.082, 0.136) in 2019. There were notable fluctuations for the period between 1990 and 2003. The snail density declined steadily from 2003 (1.142/0.11 m^2^, 95% *CI:* 0.765, 1.707) to 2012 (0.127/0.11 m^2^, 95% *CI:* 0.081, 0.199). Between 2012 and 2019, the density fluctuated slightly and maintained lower than 0.150/0.11 m^2^. Three inflection points were identified by Joinpoint model in the downstream area: 1998, 2003, and 2012. However, only the inflection of 2003 was statistically significant, and the snail density showed a significant downward trend from 2003 to 2012 with an APC being − 20.56% (95% *CI:* − 24.15, − 16.80) (Fig. 3A).

For the upstream area of the TGD (hilly region) (Fig. 2B), the snail density decreased from 0.451/0.11 m^2^ (95% *CI:* 0.241, 0.844) in 1990 to 0.115/0.11 m^2^ (95% *CI:* 0.079, 0.167) in 2019. Before 2005, the snail density fluctuated but was generally greater than 0.300/0.11 m^2^, and peaked in 2005 (0.760/0.11 m^2^, 95% *CI:* 0.479, 1.207). The snail density declined since 2006, and was generally lower than 0.150/0.11 m^2^ between 2011 and 2019. No inflection point was identified based on the Joinpoint model, and the snail density showed a significant downward trend from 1990 to 2019 with an APC being − 6.05% (95% *CI:* − 7.97, − 7.09) (Fig. 3B).

### Spatial distribution of snail density

Between 2015 and 2019, a total of 349 independent survey locations (villages or beaches) were identified, and 125 of them had the snail density of zero at some of the sampling times. There were 21 localities where snail density was from 0.001/0.11 m^2^ to 0.020/0.11 m^2^, 55 localities with snail density ranged from 0.021/0.11 m^2^ to 0.100/0.11 m^2^, 76 localities with snail density ranged from 0.101/0.11 m^2^ to 0.300/0.11 m^2^, 40 localities with snail density ranged from 0.301/0.11 m^2^ to 0.500/0.11 m^2^) and 31 localities with snail density more than 0.500/0.11 m^2^, respectively (Additional file 1: Fig. S2).

IDW showed that snails were concentrated along the Yangtze River in the lake and marshland regions and dispersed in the mountain region, with a higher snail density in the downstream area of the TGD than the upstream area between 2015 and 2019. The downstream area with a higher snail density (> 0.101/0.11 m^2^) existed in Poyang Lake, Dongting Lake, Jianghan Plain, and the Anhui branch of the Yangtze River, and the upstream area with a higher snail density (> 0.021/0.11 m^2^) existed in the Chengdu plain, Daliang Mountain area of Sichuan, and the north-west area of Yunnan (Additional file 1: Fig. S2).

## Discussion

This study used meta-analysis to explore the dynamics of snail density over a 30-year period as well as the changing trend. The snail density demonstrated a fluctuating downward trend between 1990 and 2019, which is mainly due to the continuous promotion of snail control. The considerable changes in snail density were in line with major environmental changes such as TGD operation in 2003, devastating floods in 1991 and 1998.

Our study demonstrated that the year 2003 was a significant turning point for the snail density change in the downstream area of TGD. The APC of snail density in the downstream area was − 20.56% (95% *CI:* − 24.15, − 16.80) between 2003 and 2012, significantly higher than that in other time periods, while the snail density showed an upward trend in the upstream area between 2003 and 2005. Similar results were found that the snail density was decreasing after the TGD operation [37–39]. Considerable changes have been observed in water level, weed, and silt sediment after the TGD operation [40–42], which further impacted the ecological environment of snail survival [43]. After TGD was put into operation, the lowering of water levels during flood seasons and the early water recession in autumn would lead to a change of the microenvironment of most bottomlands [44, 45]. As a result, the bottomland, especially in the middle and high elevation, was no longer conducive to the growth of reeds, which reduced the snail survival rate [46]. In addition, low water levels destroyed "winter-land and summer-water" characteristics that were suitable for snail breeding [47, 48]. The TGD has deteriorated the ecological conditions for snail survival and reproduction and compressed their living space, resulting in a fast decrease in snail density.

This study demonstrated that the snail density in the downstream area of TGD decreased for the first two years after a flood (i.e., floods in 1991 or 1998), then increased. These results were in accordance with the longitudinal observation of snail density after the flood in the middle and lower reaches of the Yangtze River [50, 51]. The survival and breeding of snails in the marshland not only depend on suitable climate and abundant food [52] but are also closely associated with the flooding [53, 54]. Submergence during summer is essential for the growth of newly hatched snails but has detrimental effects on the ability of adult snails to survival [55, 56]. The continuous high-level water, as well as the relatively long flooding duration, was not conducive to the survival of adult snails [57]. Most snails cannot survive in fast-flow water, and sediment deposition may result in the death of snails [50]. Despite having a limited ability of mobility, snails can spread over a great distance along with floating objects during a flood. Snail can spread to snail-free or snail-eliminated environments, which may result in the appearance of newly breeding habitats [27, 58]. Therefore, the decline in the first two years after a flood can be attributed to the high mortality of adult snails and the spread of snails to newly emerging and re-emergent habitats [58]. The subsequent recovery in the third year may be due to the growth and development of new young snails.

This study also observed that the snail density decreased after a drought year in the hilly region. Snails are likely to die when drought conditions are severe and frequent [14]. Severe drought caused many water sources to dry up and the soil to become dry, which was unfavorable for snail spawning and incubation [59–61]. This may explain the temporary decline of the snail density after the drought year of 2006 or 2010.

With respect to the snail density threshold (0.020/0.11 m^2^) that blocks schistosomiasis transmission, a snail density greater than that is considered to be capable of transmitting schistosomiasis [62]. Our study found that the infested areas with a higher snail density mainly existed in Poyang Lake, Dongting Lake, Jianghan Plain, and the Anhui branch of the Yangtze River. The aforementioned areas in the middle and lower reaches of the Yangtze River form a healthy grassland wetland ecosystem for snail breeding due to the seasonal and cyclical changes in water levels [63–65]. Snail density in the mountainous region is generally lower than that in the marshland, but the snails are primarily found in ditches and rice fields in hilly regions [66]. Therefore, snail detection and molluscicide are relatively complicated due to the unique natural environment and the low economic status [67], making it easy for snail density to rebound. "Health China 2030" indicates the degree of vector control is a crucial sign of a healthy city or village [68, 69]. Due to the impact of extreme weather events and water conservancy, controlling the snail density below the threshold for spreading schistosomiasis remains a significant challenge, especially in local areas with a higher snail density [68, 69]. The findings imply the focus of snail control, and it is recommended to strengthen snail monitoring and control in the focus area.

This study has some limitations. First, this study did not include other influencing factors of snail density. Second, spatial interpolation can only indicate the key area of snail density on a large scale. Further study is needed to build correlation models that include meteorological, hydrological, vegetation, and other determinants to predict fine-scale snail density.

## Conclusions

Our study provided strong evidence that the snail density exhibited a fluctuating downward trend in the Yangtze River Basin between 1990 and 2019. In the downstream area, the operation of TGD accelerated the decline of snail density during the first decade period, then snail density fluctuated at a relatively low level. The areas with a high snail density existed in Poyang Lake, Dongting Lake, Jianghan Plain, and the Anhui branch of the Yangtze River in recent years. Long-term control and monitoring of snails need to be insisted on and strengthened.

## Supplementary Information

Additional file 1: Figure S1. Spatial distribution of the sample points between 1990–2019. Figure S2. Mean snail density between 2015 and 2019.

## Data Availability

The datasets used and/or analyzed during the current study are available from the corresponding author on reasonable request.

## Abbreviations

TGD: Three Gorges Dam
APC: Annual percentage change
*I*^*2*^: Higgins inconsistency statistic
IDW: Inverse distance weighted
DALY: Disability-adjusted life year
