# Supplementary material for: Three Gorges Dam: the changing trend of snail density in the Yangtze River basin between 1990 and 2019
Source: Infect Dis Poverty. 2023 Apr 28;12:45. doi: 10.1186/s40249-023-01095-y (PMC10142781; doi:10.1186/s40249-023-01095-y)
Supplement: Supplementary file 1 — Additional file 1: Figure S1. Spatial distribution of the sample points between 1990–2019. Figure S2. Mean snail density between 2015 and 2019. [file 40249_2023_1095_MOESM1_ESM.docx]

Supplementary materials

Appendix


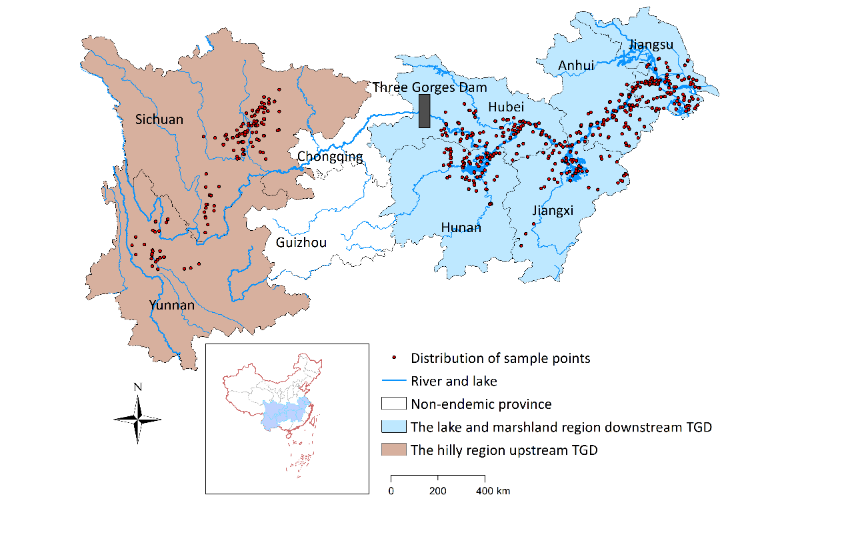


Fig. S1 Spatial distribution of the sample points between 1990-2019


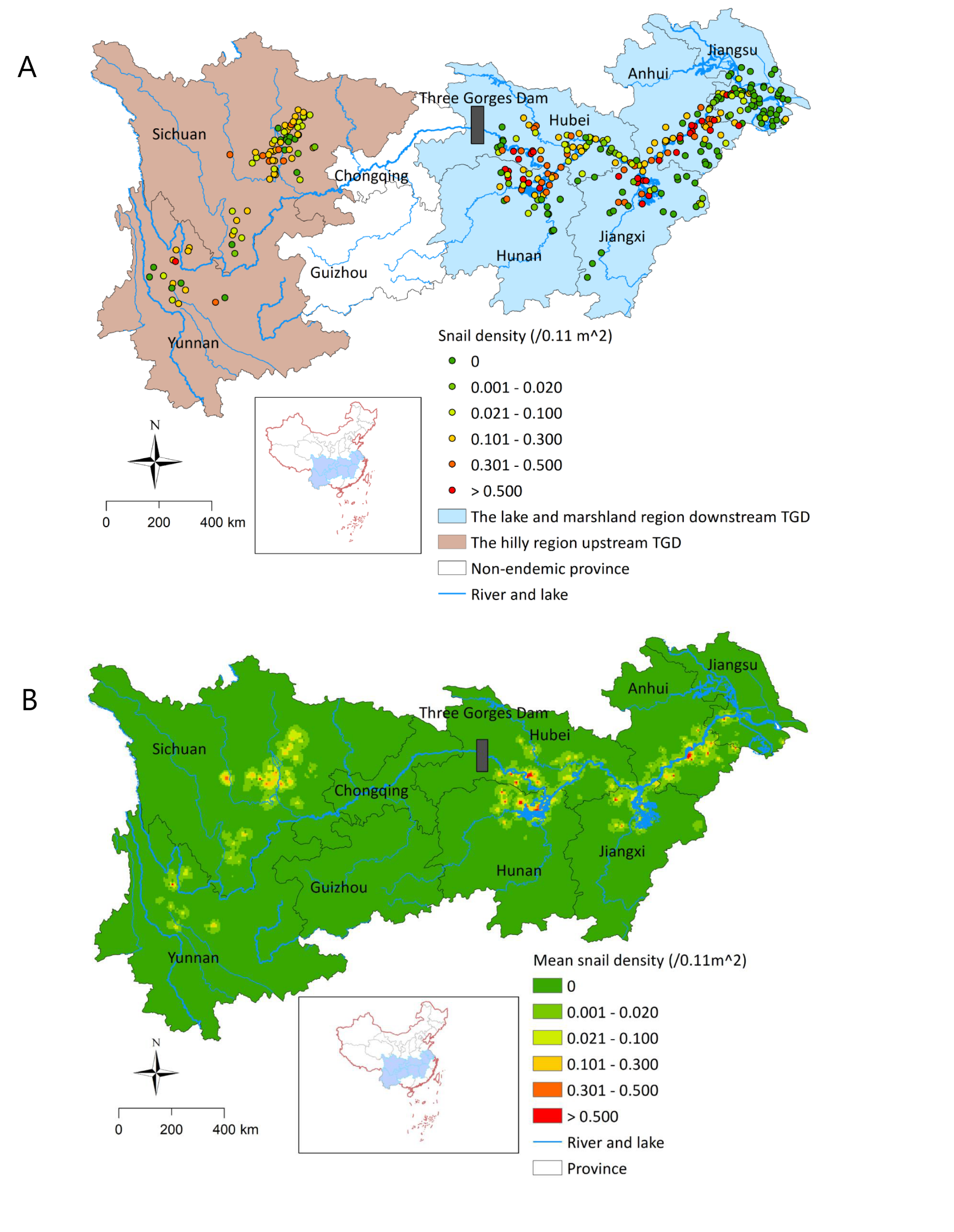


Fig. S2 Mean snail density between 2015 and 2019 (A: averaged snail density of survey point; B: mean snail density based on inverse distance weighted interpolation)
